# Localized Surface Plasmon Resonance of Silver Nanotriangles Synthesized by a Versatile Solution Reaction

**DOI:** 10.1186/s11671-015-1058-1

**Published:** 2015-09-04

**Authors:** Chunfang Wu, Xue Zhou, Jie Wei

**Affiliations:** Institute of Functional and Environmental Materials, School of Physical Science and Technology, Lanzhou University, Lanzhou, Gansu 730000 China; Electronic Materials Research Laboratory, Key Laboratory of Ministry of Education, Xi’an Jiaotong University, Xi’an, Shanxi 710049 China

**Keywords:** Silver nanotriangles, Localized surface plasmon resonance

## Abstract

The surface plasmon resonance (SPR) of silver nanoparticles can be tuned throughout the visible and near-infrared region by their shape and size. Considering SPR applications, an easy and controllable method for preparing the silver nanocrystals with defined shape and size, is necessary. In this work, the triangular silver nanoplates were synthesized by reducing Ag^+^ ions with ascorbic acid in the presence of silver seeds and poly(vinylpyrrolidone) (PVP) at room temperature. Both the seeds (as the nucleation sites) and PVP (as the capping reagent) played an important role in determining the edge length of the silver nanotriangles. The SPR of silver nanotriangles showed three distinct bands corresponding to the in-plane dipole, quadrupole, and out-plane quadrupole plasmon resonance, and the SPR shifted to shorter wavelengths with the decreased edge length of the silver nanotriangles as the theoretical calculation.

**Electronic supplementary material:**

The online version of this article (doi:10.1186/s11671-015-1058-1) contains supplementary material, which is available to authorized users.

## Background

The metal nanocrystals have attracted steadily growing attention due to their fascinating optical and electrical properties. The special optical behavior of metal nanocrystals results from the interaction of their free conduction electrons with the incident light. When the incident electromagnetic wave interacts with metal nanoparticles, localized surface plasmon resonance (LSPR), that is a collective oscillation of conduction electrons, is excited [1, 2]. In the far field, LSPR leads to absorption and scattering of the incident electromagnetic wave which is shown by the emergence of an intense band in the extinction spectrum. In the near field, intense local electromagnetic fields within a few nanometers of a particle surface are generated. This near-field effect has been gaining attention in areas that include surface-enhanced Raman scattering (SERS), plasmon-enhanced catalysis and photocatalysis, plasmon-enhanced fluorescence, the development of plasmonic solar cells, plasmonic solar water splitting process, and biological imaging and sensing [3–12].

Among all metal nanoparticles, silver nanoparticles are particularly interesting because silver has the strongest plasmonic interaction with light. As a matter of fact, the scattering cross-section of Ag is greater than that of the other kinds of metals [13]. Moreover, silver displays sharper LSPR bands which are desirable for the plasmonic application in sensors. The LSPR peak wavelength of the silver nanoparticles can be tuned throughout the visible and near-infrared region by their shape, size, and dielectric environment. Compared with spherical or quasi-spherical silver nanoparticles, the anisotropic morphology such as nanotriangles will show more LSPR bands with their decreased symmetry and the in-plane dipole resonance can be easily tailed by their edge length [14]. Furthermore, many theoretical models [15–17] and experiments have shown that nanotip provides not only the large local field enhancement but also high spatial resolution since most signals are generated from the tip area.

For the past decade, many authors reported the solution-phase methods for preparing the silver nanotriangles [18–26]. One of the most often used methods is the production of silver nanotriangles from Ag^+^ salts solution by exposure to UV-vis radiation or via chemical reduction. For instance, Mirkin and coworkers [18, 19] used the photo-induced procedure to transform small silver nanospheres into nanotriangle plates. Chen [20] prepared the silver nanotriangles by reduction of silver ions with ascorbic acid on silver seeds in the presence of micelles of cetyltrimethylammonium bromide (CTAB) which served as a soft template. Furthermore, the silver nanotriangles were synthesized by boiling silver nitrate dissolved in N,N-dimethyl formamide (DMF) which also served as the reducing agent in the presence of poly(vinylpyrrolidone) (PVP) [23]. Most recently, Liu [24] synthesized silver nanotriangles in one step by an elaborately designed coordination-based kinetically controlled seed growth. However, from the point of view for application, an easy, green, and controllable approach which could define the relationship between the edge length of the silver nanotriangles with the reaction conditions is scarce and needed to be developed. As known that with the aid of the oxygen and nitrogen atoms of the pyrrolidone unit, PVP chains can adsorb onto the surface of silver as a capping agent or stabilizer. The interaction strength between PVP and different crystallographic facets of a silver lattice was substantially different and could therefore induce anisotropic growth for silver nanoparticles. Both Wiley [27] and Kan [28] reported the preparation of silver nanocubes and nanowires by a PVP-mediated polyol process. Gao’s works [29, 30] showed silver nanowires and nanodecahedrons could be achieved with the structure-directing effect of PVP. Nevertheless, further studies on the other anisotropic shape of silver nanocrystals resulted from the PVP-mediated morphological evolution are required. Herein, we described a seed-mediated silver nanotriangle growth process with a different edge length and a tunable LSPR band that involved the use of PVP. A set of experiments were designed to establish the roles of seeds and PVP on the nanotriangles formation and the edge length in this solution reaction.

## Methods

### Materials

The materials used were as follows: silver nitrate (99.8 %, AR), sodium borohydride (98 %), trisodium citrate (99.0 %, AR), ascorbic acid (>99.0 %, AR), and poly(vinylpyrrolidone) (K85-95, *M*_w_ = 1,300,000). All chemicals were purchased from the Aladdin Industrial Corporation and used without any further purification. All aqueous solution were prepared using ultrapure water with a resistivity of 18 MΩ∙cm.

### Nanotriangles Preparation

In the first step, 20 mL stock aqueous solution of silver particles called ‘seeds’ with a diameter of about 4 nm was prepared through rapid reduction reaction of 0.85 mg silver nitrate by 1 mL sodium borohydride (1 M) in water with 2.94 mg trisodium citrate as the stabilizer. In the second step, 100 mL silver nitrate (2.5 mM), PVP, and seeds solution were mixed together. Then, 20 mL of ascorbic acid (0.1 M) was added dropwise under continuous stirring. The edge length of silver nanotriangles was tuned by the ratio between capping agent (PVP) and the precursor salt (AgNO_3_) as well as the dosage of the seeds solution such as 10 μL, 100 μL, or 1 mL. All these reactions proceeded at room temperature. The products were obtained by centrifugation for three times and redispersed in alcohol.

### Nanotriangles Characterization

UV-visible-near-infrared (NIR) absorption spectrometer was measured in a 1-cm-quartz cuvette with an optical path of 1 cm using a PerkinElmer Lambda 950 spectrophotometer. A drop of the alcoholic suspension of silver nanoparticles was placed on a carbon-coated copper grid and dried under an infrared lamp for transmission electron microscope (TEM) characterization. TEM images were captured using a FEI Tecnai F30 transmission electron microscope. X-ray powder diffraction (XRD) were performed using a Bruker D2 phaser with Cu Kα radiation (*λ* = 0.154056 nm).

## Results and Discussion

**Fig. 1:**
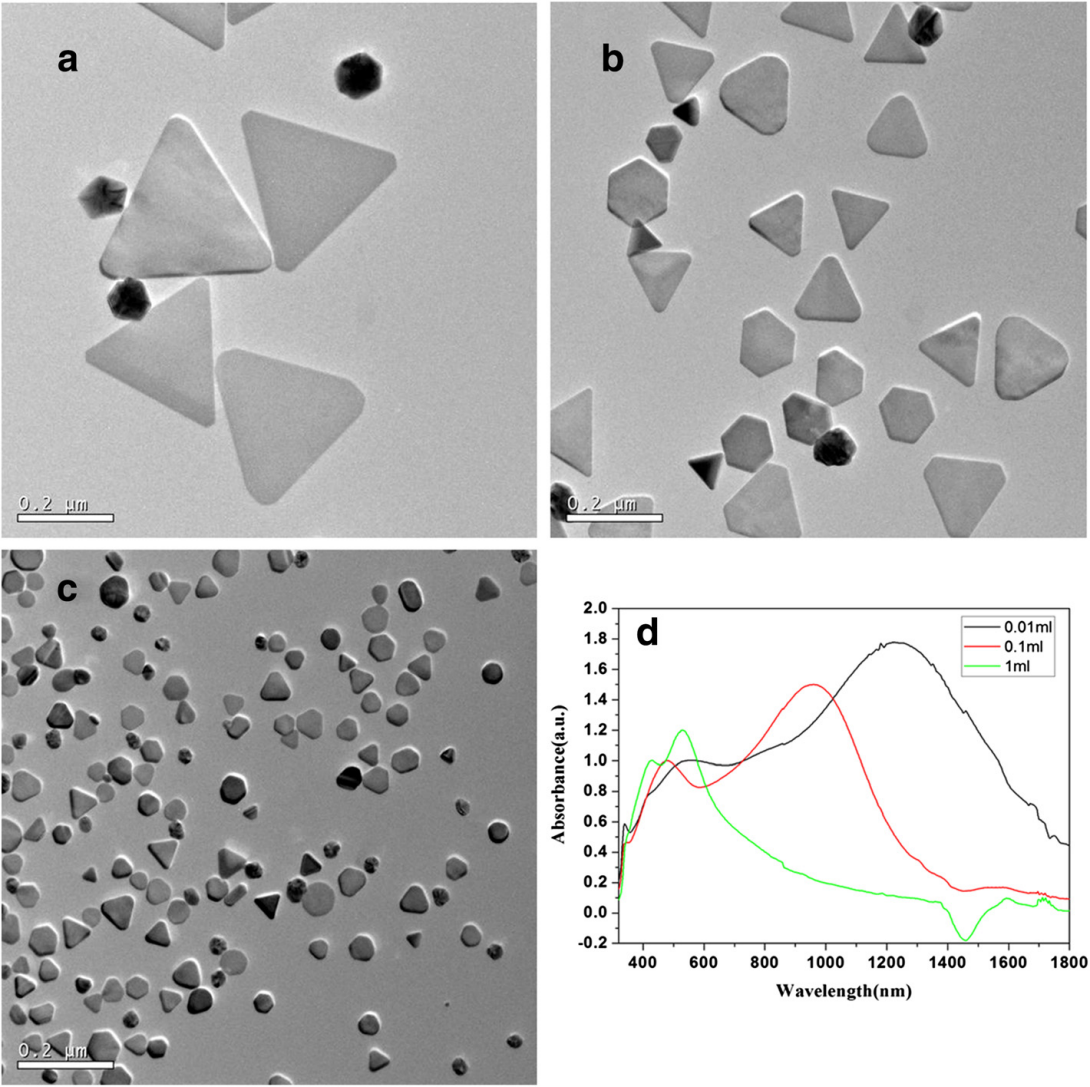
TEM images and absorption spectra of as-prepared silver nanotriangle with different dosage of seeds solution. **a** 0.01 mL. **b** 0.1 mL. **c** 1 mL. **d** Vis-NIR absorption spectra of the corresponding samples

Figure 1a–c shows the TEM images taken from the silver nanoparticles that were synthesized with adding three different volume seeds solution while the molar ratio of PVP (calculated in terms of the repeating unit) to AgNO_3_ was kept as 2. For convenience, the scale bar in the three TEM micrographs is kept the same (the large area TEM, shapes distributions histogram, and the corresponding size distribution were arranged in the Additional file 1: Figure S1, ESI). The average edge length of the silver nanotriangles (listed in Table 1, ranged from 50 to 260 nm) decreased with the dosage of seeds solution increasing. Meanwhile the nanotriangles gradually turned truncated, hexagonal nanoplates, even the circular nanodisks with increasing the dosage of seeds solution. Under the same amount of ingredient for crystals to grow, the more seeds concentration indicates the more nucleation sites and the shorter size of the nanocrystals [31]. A relationship between the edge length (*L*) and the volume of the seeds solution (*V*), *L*^2.437^ = k*V*^−1^(*k* is a constant), was summarized in Liu’s work [24]. The shape evolution probably derived from the fact that the vertex atoms of the nanotriangles are more labile than other surface atoms, and they will migrate to the sides of the triangle to lower the surface energy [32].

**Table 1:** Relationship between the edge length of silver nanotriangles and the plasmon resonance position

Samples	Average edge length (nm)	Plasmon resonance position (nm)		
		In-plane dipole	In-plane quadrupole	Out-of-plane quadrupole
0.01 mL	260	1234	544	339
0.1 mL	130	955	478	340
1 mL	50	528	428	–
PVP1	217	1129	534	340
PVP2	130	957	480	338
PVP4	100	792	440	–

The size-dependent surface plasmon resonance (SPR) properties of the silver nanotriangles were shown in Fig. 1d. The spectra of the silver nanotriangles showed three dominant peaks corresponding to different modes of plasmon excitation. Considering the spectrum of the nanotriangles prepared with 0.01 mL seeds solution, the strong bands located at 1234 and 544 nm are ascribed to the in-plane dipole and quadrupole plasmon resonance. The in-plane dipole and quadrupole plasmon resonances (listed in Table 1) were red-shifted as increasing the edge length of the nanotriangles from 50 to 260 nm. This red-shift can be assigned to the increased charge separation during plasmon oscillation as enabled by the increase in size. The bluest resonance band at 339 nm (or 340 nm), which was the out-of-plane quadrupole resonance, was nearly invariable despite the variation of the edge length of the triangles. Also, the width of the peaks is larger in comparison with the theoretical spectra [15–17]. This result can be explained by the presence in the colloidal solution of a certain percent of silver particles with different shape and size which contributed to an inhomogeneous damping of the spectra.

**Fig. 2:**
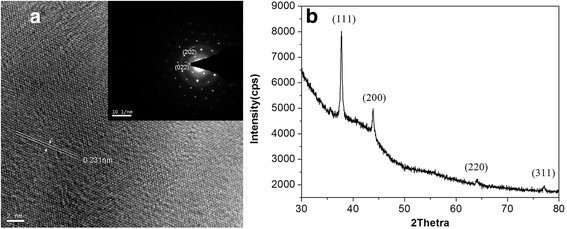
HR-TEM images (**a**) and XRD (**b**) of silver triangular nanoplates

The structure of the nanotriangles prepared with 0.01 mL seeds solution was further characterized using the high-resolution TEM image and the X-ray diffraction patterns. Figure 2a shows the HR-TEM image of the nanotriangles. The fringes are separated by 2.31 Å which can be ascribed to the reflection of (111) crystal face. The inset in Fig. 2a shows a typical electron diffraction pattern recorded by directing the electron beam along the [111] zone axis which is perpendicular to the triangular flat faces of an individual nanoplate. The spot points with a hexagonal arrangement clearly indicate that the particle is a single crystal and can be indexed to the $\left\{20\overline{2}\right\}$ Bragg reflection of face-centered cubic silver. According to the standard diffraction pattern of [111] zone axis of face-centered cubic (fcc) structure, the crystal face index of two adjacent spot points can be obtained as $\left(02\overline{2}\right)$ and $\left(20\overline{2}\right)$. The index of other spot points also can be obtain by addition or subtraction of the corresponding two reciprocal vectors.

The formation of fcc silver crystals was also confirmed by the XRD pattern. From Fig. 2b, we can see four Bragg diffraction peaks at 37.8°, 43.9°, 64.1°, and 77.1° which pertain to (111), (200), (220), and (311) planes of silver (JCPDS, card No. 04-0783). The exceeding high diffraction peak of (111) plane over other three peaks clearly revealed that the sample exclusively comprised the nanoplates that were preferentially oriented with their (111) planes parallel to the supporting substrate.

The above structure analysis clearly demonstrates that the basal plane, i.e., the top crystal plane of the triangular plates, should be the (111) plane. We can see that this feature is quite common for the metal nanoplates [33, 34]. A general order of the surface energies for different faces of the fcc metals may hold [35], *γ*{111} < *γ*{100} < *γ*{110}. That is, more energy is released by adding a silver atom to the {100} faces or the {110} faces, rather than the {111} faces during crystal growth. As a result, crystal growth can be accelerated by biasing accretion onto {100} faces, {110} faces, or others, thus increasing the area of the {111} faces.

**Fig. 3:**
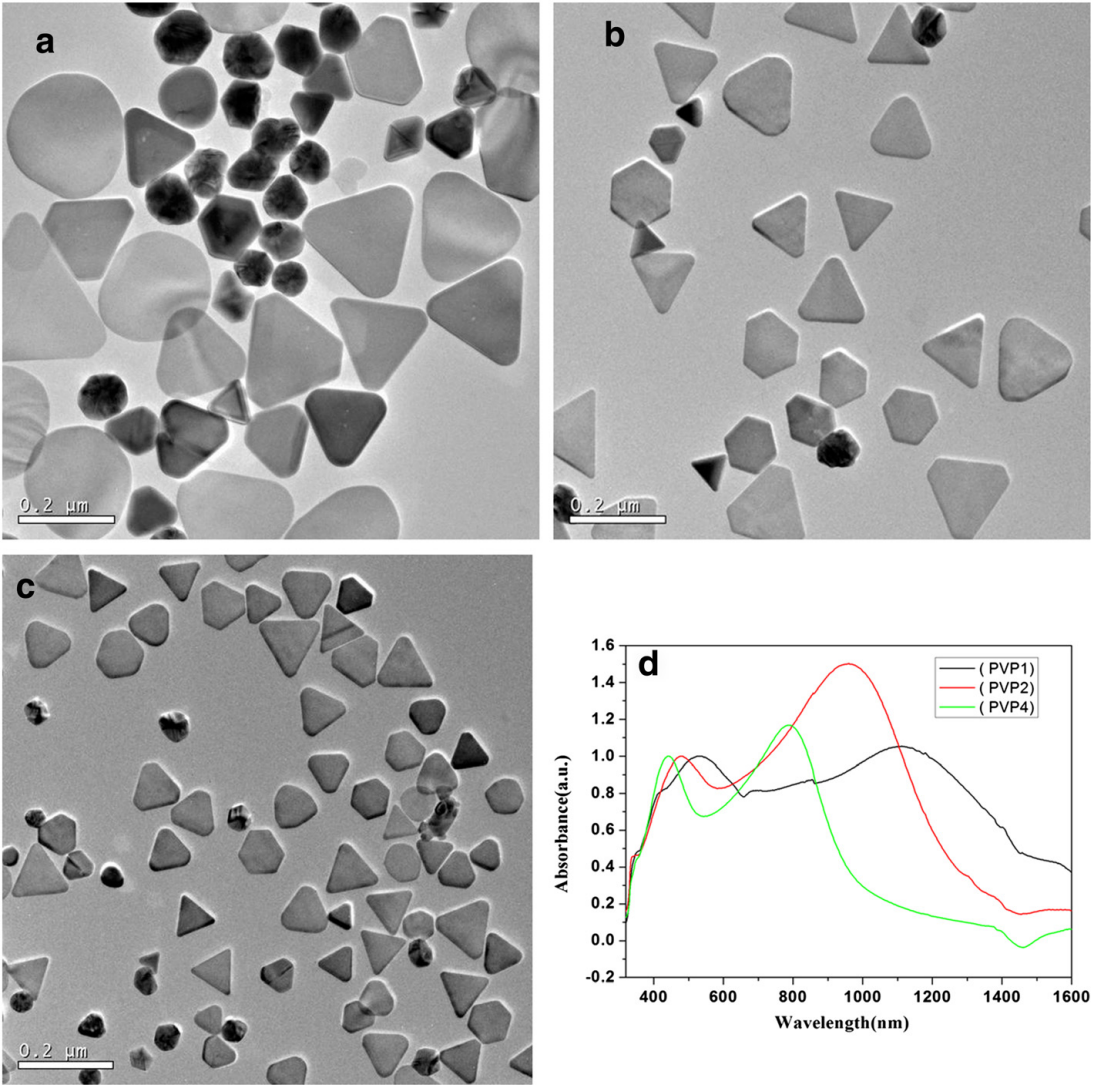
TEM images and absorption spectra of as-prepared silver nanotriangles with different molar ratios between PVP and AgNO_3_, **a** 1, **b** 2, **c** 4. **d** Vis-NIR absorption spectra of corresponding samples

Besides the dosage of seeds solution tuning the edge length of silver nanotriangles, the different dosage of PVP also has this kind of function in the series reactions. The morphology of the nanocrystals prepared by adjusting the molar ratio between PVP and AgNO_3_ as 1, 2, and 4 with adding 0.1 mL seeds solution was shown in the Fig. 3. The large area TEM, shape distribution histogram, and the corresponding size distribution were arranged in Additional file 1: Figure S2, ESI. The truncated triangles were obtained, and the edge length decreased with increasing the molar ratio between PVP and AgNO_3_. This phenomenon also occurred in the reaction of producing golden nanoplate [36]. It is suggested that PVP is selectively capped on the {100} rather than on the {111} or {110} facets of Ag seeds. Therefore, the coverage percentage of PVP on the surface of seeds has a linear dependence on the molar ratio of PVP to AgNO_3_. At a high PVP concentration, large amounts of PVP molecules were selectively bound on the basal {100} facets of the silver seeds, the growth rate of silver nanocrystals decreased with the thick PVP layer hindering the silver ions in reaching the growing plane. Moreover, the high PVP concentration also resulted in an increased viscosity of solution and a low transfer rate of silver ions toward the silver seeds, which, therefore, impeded the growth of the silver crystals [37]. The decreased size leads to the blue shift of the SPR as shown in the Fig. 3d. As a referred sample, the in-plane dipole resonance data of sample prepared under the molar ratio of PVP to AgNO_3_ as 4 was compared with the calculation from the discrete dipole approximation method (DDA) and the finite difference time domain method (FDTD). The corresponding data are listed in Table 2. It was reported that the SPR position was affected by the edge length exclusively in Shi’s [15] calculation, or affected by the aspect ratio (which is defined as the ratio of the edge length to the thickness of the nanoprism) in Pileni’s [16] work. Whatever, the experimental data of our work agreed closely with those theoretically derived spectra.

**Table 2:** Comparison of in-plane dipole resonance of silver triangles between experimental data and theoretical calculation

Average edge length (nm)		Simulated results (nm)			
	Our work	DDA [15]	DDA [38]	DDA [17]	FDTD [39]
100	792	674 (10^a^)	770 (16^a^)	830 (10^a^)	760 (15^a^)

^a^Denote the thickness of the nanotriangle

## Conclusions

The method demonstrated in this work provided a simple, reproducible, controllable route to synthesize silver triangles. By adjusting the amount of the seeds, or the mole ratio between PVP and AgNO_3_, the edge length of the silver triangles can be conveniently tuned in the range from 50 to 260 nm. The surface plasmon resonance regularly varied with the edge length of the silver nanotriangles. With optimized conditions, such as slower dropping speed and suitable concentration of the trisodium citrate, the silver triangles with sharper corner and narrower size distribution will be synthesized.

## Additional file

Additional file 1:
**The size and the shape distribution histogram of the as-prepared silver nanoparticles.** **Figure S1.** TEM images (a, b, c), the different nanoparticles shapes distributions histogram (d, e, f) and the corresponding edge length distributions histogram (g, h, i) of as-prepared silver nanotriangles with different dosages of seeds solution. (a), (d), and (g) for 0.01 mL; (b), (e), and (h) for 0.1 mL; (c), (f), and (i) for 1 mL. In (d), (e), and (f) the T-N, Q-N, T-T, and R-N are the abbreviations for triangular nanoparticles, quasi-spherical nanoparticles, truncated triangles, and rounded nanoplates. **Figure S2.** TEM images (a, b, c), the different nanoparticles shapes distributions histogram (d, e, f), and the corresponding edge length distributions histogram (g, h, i) of as-prepared silver nanotriangles with different molar ratio of PVP to AgNO_3_. (a), (d), and (g) for 1. (b), (e), and (h) for 2. (c), (f), and (i) for 4. In (d), (e), and (f) the T-N, Q-N, T-T and R-N are the abbreviations for triangular nanoparticles, quasi-spherical nanoparticles, truncated triangles, and rounded nanoplates. **Figure S3.** Absorption spectra of products obtained with the seeds solution dosage as 0.01, 0.1, and 1 mL. **Figure S4.** Absorption spectra of products obtained when the molar ratio between PVP and AgNO_3_ as 1, 2 and 4.
