# Supplementary material for: Localized Surface Plasmon Resonance of Silver Nanotriangles Synthesized by a Versatile Solution Reaction
Source: Nanoscale Res Lett. 2015 Sep 4;10:354. doi: 10.1186/s11671-015-1058-1 (PMC4560725; doi:10.1186/s11671-015-1058-1)
Supplement: Additional file 1: — The size and the shape distribution histogram of the as-prepared silver nanoparticles. Figure S1. TEM images (a, b, c), the different nanoparticles shapes distributions histogram (d, e, f) and the corresponding edge length distributions histogram (g, h, i) of as-prepared silver nanotriangles with different dosages of seeds solution. (a), (d), and (g) for 0.01 mL; (b), (e), and (h) for 0.1 mL; (c), (f), and (i) for 1 mL. In (d), (e), and (f) the T-N, Q-N, T-T, and R-N are the abbreviations for triangular nanoparticles, quasi-spherical nanoparticles, truncated triangles, and rounded nanoplates. Figure S2. TEM images (a, b, c), the different nanoparticles shapes distributions histogram (d, e, f), and the corresponding edge length distributions histogram (g, h, i) of as-prepared silver nanotriangles with different molar ratio of PVP to AgNO3. (a), (d), and (g) for 1. (b), (e), and (h) for 2. (c), (f), and (i) for 4. In (d), (e), and (f) the T-N, Q-N, T-T and R-N are the abbreviations for triangular nanoparticles, quasi-spherical nanoparticles, truncated triangles, and rounded nanoplates. Figure S3. Absorption spectra of products obtained with the seeds solution dosage as 0.01, 0.1, and 1 mL. Figure S4. Absorption spectra of products obtained when the molar ratio between PVP and AgNO3 as 1, 2 and 4. [file 11671_2015_1058_MOESM1_ESM.docx]

Electronic Supplementary Information (ESI)

**Localized surface plasmon resonance of silver nanotriangles synthesized by a versatile solution reaction**

Chunfang Wu*^a^, Xue Zhou^a^, Jie Wei^b^

a. Institute of Functional and Environmental Materials, School of Physical Science and Technology, Lanzhou University, Lanzhou, Gansu 730000, China. E-mail: wuchf@lzu.edu.cn

b. Electronic Materials Research Laboratory, Key Laboratory of Ministry of Education, Xi’an Jiaotong University, Xi’an, Shanxi 710049, China. E-mail: weij2008@mail.xjtu.edu.cn


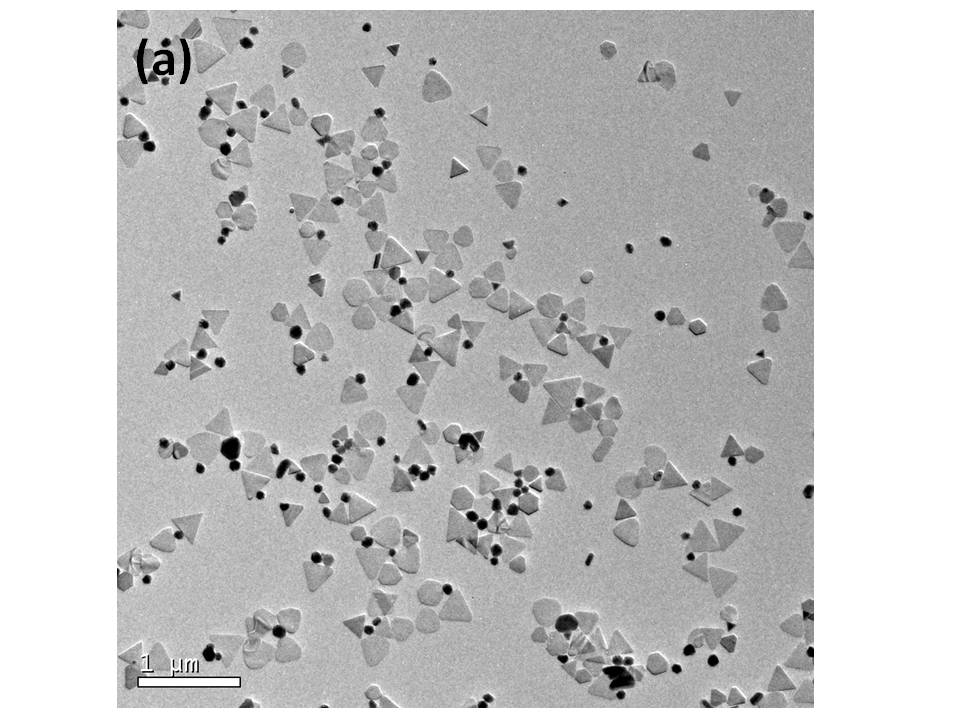


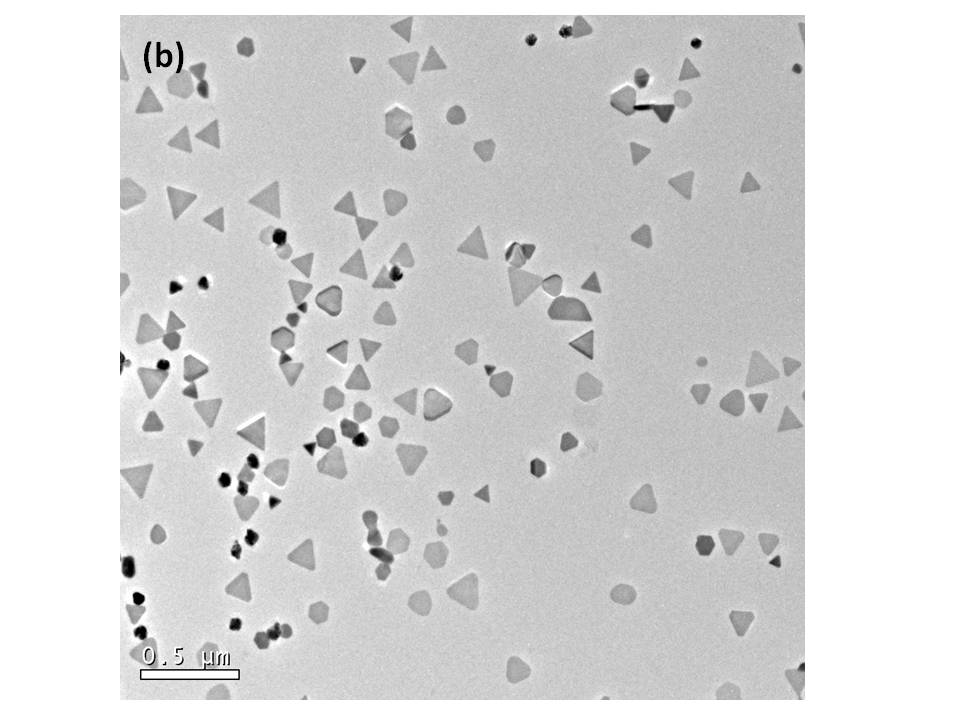


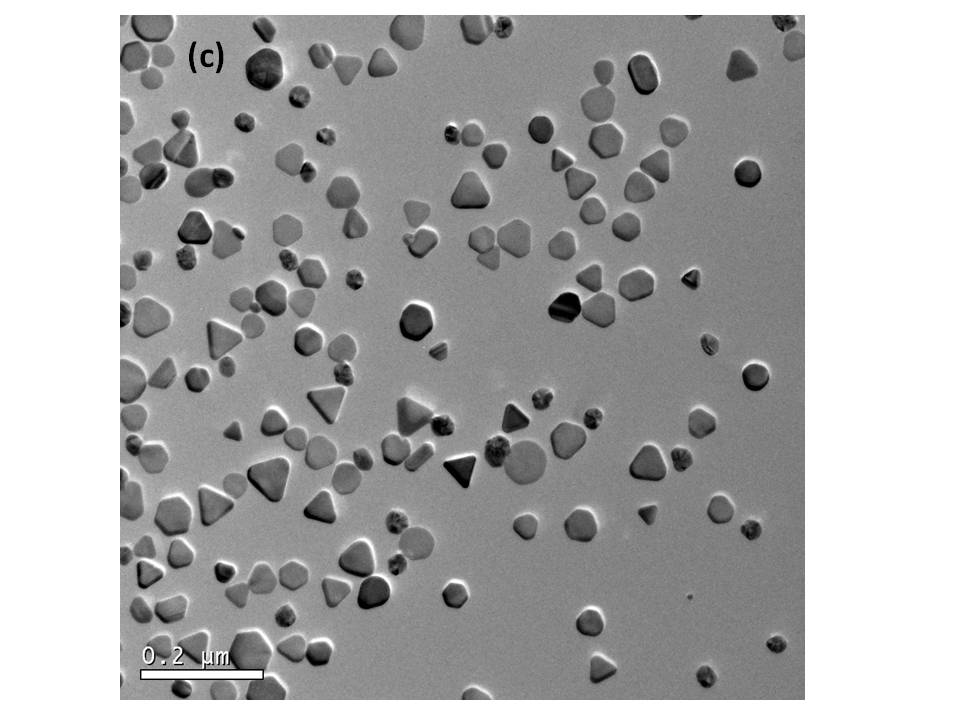


Figure S1. TEM images (a, b, c), the different nanoparticles shapes distributions histogram (d, e, f) and the corresponding edge length distributions histogram (g, h, i) of as-prepared silver nanotriangles with different dosage of seeds solution. (a), (d) and (g) for 0.01 ml. (b), (e) and (h) for 0.1 ml. (c), (f) and (i) for 1 ml. In (d), (e) and (f) the T-N, Q-N, T-T and R-N are the abbreviations for triangular nanoparticles, quasi-spherical nanoparticles, truncated triangles and rounded nanoplates.


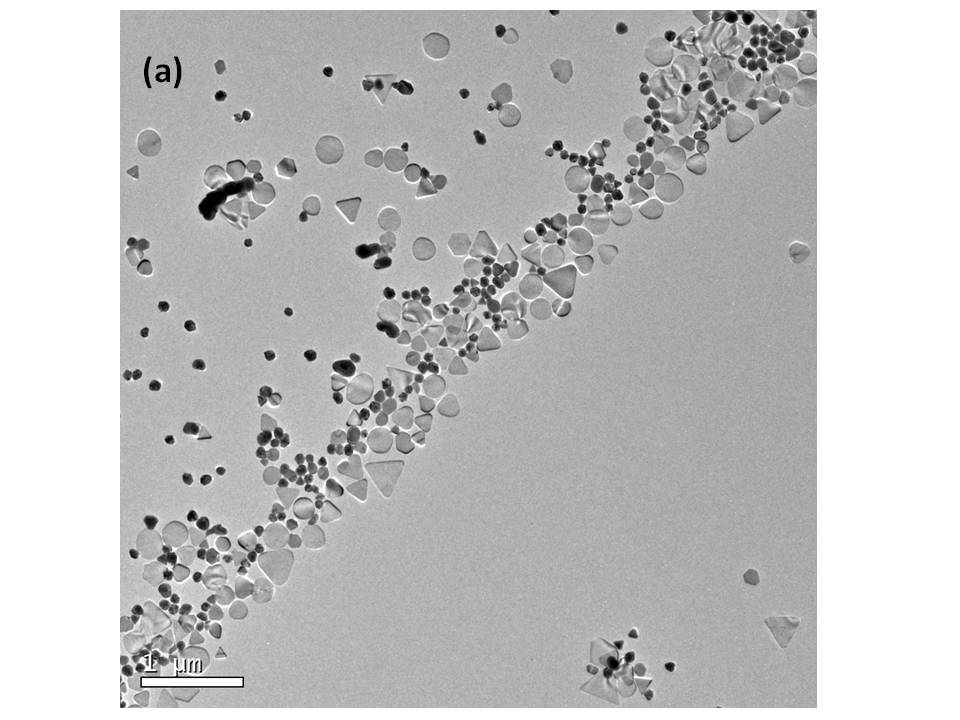


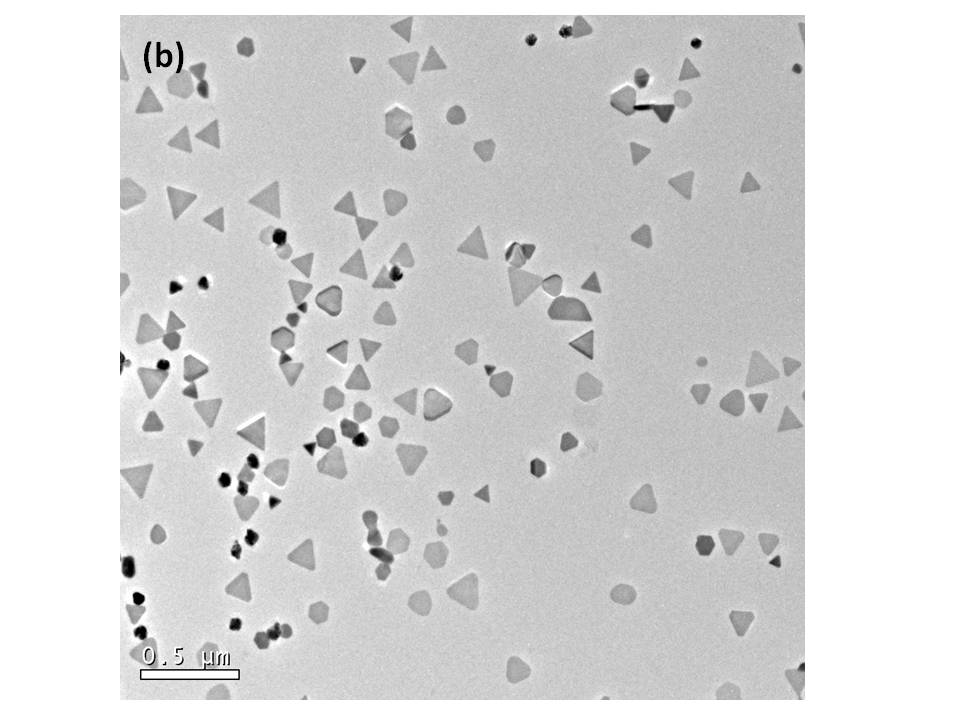


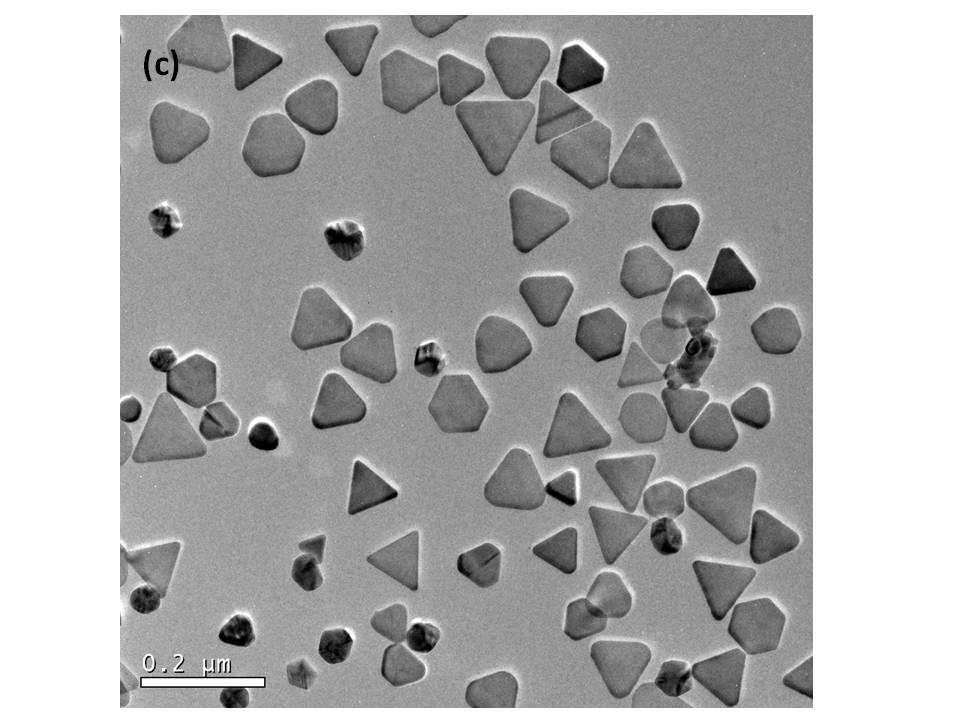


Figure S2. TEM images (a, b, c), the different nanoparticles shapes distributions histogram (d, e, f) and the corresponding edge length distributions histogram (g, h, i) of as-prepared silver nanotriangles with different molar ratio of PVP to AgNO_3_. (a), (d) and (g) for 1. (b), (e) and (h) for 2. (c), (f) and (i) for 4. In (d), (e) and (f) the T-N, Q-N, T-T and R-N are the abbreviations for triangular nanoparticles, quasi-spherical nanoparticles, truncated triangles and rounded nanoplates.

Figure S3. Absorption spectra of products obtained with the seeds solution dosage as 0.01 ml, 0.1 ml and 1 ml

Figure S4. Absorption spectra of products obtained when the molar ratio between PVP and AgNO_3_ as 1, 2 and 4
